# Natural Bio-Compounds from *Ganoderma lucidum* and Their Beneficial Biological Actions for Anticancer Application: A Review

**DOI:** 10.3390/antiox12111907

**Published:** 2023-10-25

**Authors:** Emin Cadar, Ticuta Negreanu-Pirjol, Carolina Pascale, Rodica Sirbu, Irina Prasacu, Bogdan-Stefan Negreanu-Pirjol, Cezar Laurentiu Tomescu, Ana-Maria Ionescu

**Affiliations:** 1 Faculty of Pharmacy, “Ovidius” University of Constanta, Capitan Aviator Al. Serbanescu Street, No. 6, Campus, Building C, 900470 Constanta, Romania; emin.cadar@365.univ-ovidius.ro (E.C.); bogdan.negreanu@univ-ovidius.ro (B.-S.N.-P.); 2 Academy of Romanian Scientists, Ilfov Street, No. 3, 050044 Bucharest, Romania; 3 Organizing Institution for Doctoral University Studies of “Carol Davila”, University of Medicine and Pharmacy of Bucharest, Dionisie Lupu Street, No. 37, Sector 2, 020021 Bucharest, Romania; carolina.pascale@drd.umfcd.ro; 4 Faculty of Pharmacy, “Carol Davila” University of Medicine and Pharmacy of Bucharest, Traian Vuia Street, No. 6, Sector 2, 020956 Bucharest, Romania; irina.prasacu@umfcd.ro; 5 Faculty of Medicine, “Ovidius” University of Constanta, University Alley, No. 1, Campus, Building B, 900470 Constanta, Romania; tomescu.cezar.laurentiu@gmail.com (C.L.T.); ana.maria.ionescu@univ-ovidius.ro (A.-M.I.); 6 “Sf. Ap. Andrei” County Clinical Emergency Hospital, Tomis Bvd., No. 145, 900591 Constanta, Romania; 7 Clinical Hospital C F Constanta, 1 Mai Bvd., No. 3–5, 900123 Constanta, Romania

**Keywords:** natural bio-compounds, *Ganoderma lucidum*, polysaccharides, triterpenoids, antitumor activity, immunomodulatory, antioxidant, cytotoxic

## Abstract

*Ganoderma lucidum* (*G. lucidum*) has been known for many centuries in Asian countries under different names, varying depending on the country. The objective of this review is to investigate the scientific research on the natural active bio-compounds in extracts obtained from *G. lucidum* with significant biological actions in the treatment of cancer. This review presents the classes of bio-compounds existing in *G. lucidum* that have been reported over time in the main databases and have shown important biological actions in the treatment of cancer. The results highlight the fact that *G. lucidum* possesses important bioactive compounds such as polysaccharides, triterpenoids, sterols, proteins, nucleotides, fatty acids, vitamins, and minerals, which have been demonstrated to exhibit multiple anticancer effects, namely immunomodulatory, anti-proliferative, cytotoxic, and antioxidant action. The potential health benefits of *G. lucidum* are systematized based on biological actions. The findings present evidence regarding the lack of certainty about the effects of *G. lucidum* bio-compounds in treating different forms of cancer, which may be due to the use of different types of *Ganoderma* formulations, differences in the study populations, or due to drug–disease interactions. In the future, larger clinical trials are needed to clarify the potential benefits of pharmaceutical preparations of *G. lucidum*, standardized by the known active components in the prevention and treatment of cancer.

## 1. Introduction

*Ganoderma lucidum* (*G. lucidum*), (Fr.) Karst is a medicinal mushroom known in traditional Asian medicine under different names depending on the country: Lingzhi (China), Reishi, and Mannentake (Japan), Linh chi (Vietnam), and Yeong Ji or Yung Gee (Republic of Korea) [1]. It is considered a source of longevity and health promotion [2,3,4]. The traditional medicinal uses of these mushrooms in Chinese and Japanese folk medicine as health remedies and herbal supplements are widely recognized [5,6,7]. The knowledge and use of medicinal mushrooms have preoccupied researchers, who have developed valuable studies on their bioactive components and their importance [8,9,10,11,12]. According to the World Health Organization, it has been estimated that around three-quarters of the world’s population relies on traditional medicines to maintain their health [13]. In traditional oriental medicine, *G. lucidum* has been used to treat several diseases, such as asthma, bronchitis, arthritis, hypertension, insomnia, diabetes, liver disease, nephritis, and cancer [14,15,16]. In ancient Chinese medicine, *G. lucidum* has been used for longevity and as an anticancer and antioxidant agent [17,18,19,20]. In the last 30 years, data have been reported on the chemical composition of *G. lucidum* extracts, justifying their biological activity and numerous health benefits. Among the most important bioactive compounds are polysaccharides and triterpenes [21,22,23,24,25,26]. Numerous other bioactive metabolites, such as proteins, peptides, sterols, lectins, adenosine, vitamins, and metals, have also been identified [27,28,29,30,31]. However, it should be noted that there are differences in the composition of *G. lucidum* products due to cultivation conditions, the extraction process of the bio-compounds, and the origin or part of the fungus used (fruiting body, mycelium, or spores) [14,28,30]. Nevertheless, many bioactive compounds in *G. lucidum* exhibit antioxidant, antitumor, and anti-inflammatory properties [32,33,34,35,36,37]. Such benefits have been investigated in multiple studies on their effect against prostate cancer, ovarian cancer, hepatocellular carcinoma, the induction of apoptosis in colon cancer, and the inhibition of angiogenesis [38,39,40,41,42,43]. Different immunomodulatory activities have been reported [44,45,46,47]. Other researchers have also reported studies on antioxidant and antioxidative stress actions [48,49,50]. With the development of modern research techniques to identify the chemical compositions of *G. lucidum* compounds, data may be accessible for use in medical research. A detailed understanding of these biological mechanisms could greatly influence and extend their benefits to human health.

The present study aims to review the data on active bio-compounds with important biological actions against cancer. Factors influencing the composition of the *G. lucidum* fungus, such as growing conditions (in the wild, in deciduous forests, or in special crops) and territorial areas, are analyzed. The characteristics of the *G. lucidum* fungus, as well as the chemo-bioactive compounds identified in *G. lucidum* with anticancer activities, its biological activities against cancer, and its toxicity and safety, are described in separate sections. The mechanisms of anticancer effects, including other biological activities that contribute to the fight against cancer, such as immunomodulatory, antioxidant, and cytotoxic actions, are also presented. Some negative cases in which *G. lucidum* bio-compounds were administered to patients undergoing treatment for several conditions are included as well. In these patients, the effects of *G. lucidum* treatments were not beneficial due to the occurrence of adverse effects.

## 2. Characteristics of *Ganoderma lucidum* Fungus

### 2.1. Description and Spread of the Fungus

**Figure 1:**
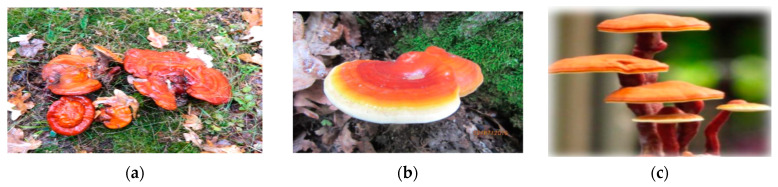
Appearance of *Ganoderma lucidum* in Europe (Romania) and Asia [61,62]. (**a**) *G. lucidum* from Neamt County, Romania, adapted from [61]. (**b**) *G. lucidum* from Bacau County, Romania, adapted from [61]. (**c**) Reishi or Lingzhi from India, adapted from [62].

The first description of *Ganoderma lucidum* (Curtis) P. Karst. was made by Curtis in England, and this description was officially recorded by Fries [51]. Initially, Cao et al. claimed that this fungus was already known as “Lingzhi”, a medicinal fungus identified and used in China for more than 2000 years [52]. Later, based on molecular studies, it was established that the East Asian medicinal mushroom is a different species from the *G. lucidum* mushroom [52]. After morphological and molecular examinations, Wang et al. confirmed that the Lingzhi species from China are related to *G. lucidum* from the UK and to other *Ganoderma* species [53]. Kwon et al. conducted phylogenetic analyses of *Ganoderma* species and showed that there are 62 strains of *Ganoderma* [54]. According to the taxonomic classification established by Nahata A., the species *Ganoderma lucidum* (Curt: Fr.) Karst belongs to the kingdom *Fungi*, phylum *Basidiomycota*, class *Agaricomycetes,* order *Polyporales*, family *Ganodermataceae*, genus *Ganoderma*, and species *lucidum* [55]. *G. lucidum* from the UK and other related *Ganoderma* species have also been morphologically and molecularly examined by different researchers who have conducted important studies, such as Kim et al., Park et al., and Liao et al. [56,57,58]. Several phylogenetically related *Ganoderma* species have been found in North America, Europe, and Asian countries [56,58]. Gottlieb et al. also performed molecular and morphological studies on the *Ganoderma* species collected from South America, followed by Eyssartier et al. from France [59,60]. In 2017, Copot et al. identified the fungus *G. lucidum* in hilly and mountainous areas in Romania, specifically in oak forests [61]. The description of *G. lucidum* collected from Romania highlighted the mushroom cap, which is kidney-shaped and can be up to 20 cm in diameter, ranging in color from red to pinkish-brown when mature [61]. Towards the edges, the coloration of the cap ranged from bright yellow to white. The spore print is brown (see Figure 1a,b) [61]. Figure 1c shows the fungus *G. lucidum* (Reishi or Lingzhi) adapted from studies by Parepalli et al. [62]. 

### 2.2. Data on G. lucidum Cultivation

**Figure 2:**
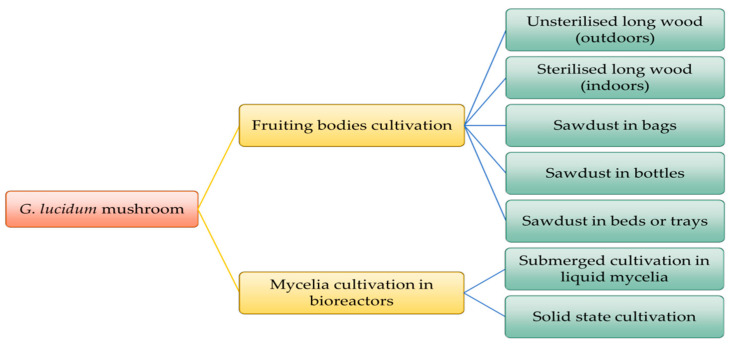
Different cultivation methods for fruit body and mycelium of *Ganoderma lucidum*, adapted from [14].

*G. lucidum* can also grow in greenhouses under controlled conditions. Data on cultivation methods have been reported in the literature, such as the study of Boh et al., where different biotechnological cultivation methods are presented [14]. As *G. lucidum* is rare in nature, cultivation in greenhouses is practiced using two methods: cultivation of fruiting bodies on wooden logs or on sawdust bags (or large plastic bottles) [14]. The main cultivation methods to produce *G. lucidum* (fruiting body and mycelia) are presented in Figure 2 [14].

Various other cultivation methods have also been practiced. For example, Nithya et al. conducted research on the selection of an ideal material to grow *G. lucidum* fungus by testing wood retting, wheat bran, sorghum, and sorghum grains combined with chalk dust and gypsum [63]. Adongbede et al. (2021) used indigenous hardwoods supplemented with rice and wheat bran as substrates for growing *G. lucidum* in Nigeria, where the fungus is not naturally available [64]. Matute et al. (2002) grew *G. lucidum* in bags using sunflower hulls as a nutrient source [65]. Yang et al. (2003) used carbohydrate and nitrogen-rich residues from a rice bran distillery to grow *G. lucidum* in polypropylene bags [66]. Hsieh et al. (2004) used soybean residues as a nutrient for growing *G. lucidum* in polypropylene bags [67]. Chang et al. (2006) reported studies on the optimization of growth methods for *G. lucidum* [68]. It is worth noting that cultivation conditions and substrate composition influence the biochemical composition of *G. lucidum* fungi qualitatively and quantitatively, as confirmed by Baskar et al. in 2011 through their studies [69].

## 3. Chemical Bioactive Compounds Identified in *G lucidum* with Anticancer Actions

In the last 30 years, numerous studies have been conducted highlighting the nutritional potential of *G. lucidum* mushrooms [30]. Research on the biochemical composition of this mushroom has led to the identification of several categories of compounds with health-promoting biological activity, as reported by Ahmad et al. [7,13].

### 3.1. Proximate Composition for G. lucidum

The values of the bioactive compound classes differ quantitatively and qualitatively within certain limits, depending on the country and area of origin of the fungus and whether it is cultivated or naturally occurring in the forest. In the case of cultivated mushrooms, bioactive compounds depend on the nutrient richness of the substrate and environmental factors, such as humidity and temperature [14]. Most phytochemical reports show that, with regard to the chemical composition, the fruiting body of *G. lucidum* consists of 90% water and 10% different compounds, as presented in Table 1, according to Mau et al. [70].

**Figure 3:**
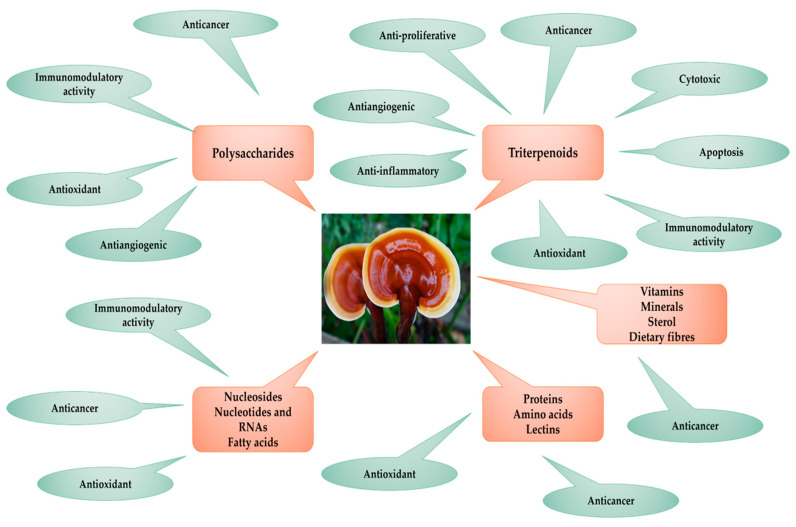
Bioactive compounds in *G. lucidum* and biological actions involved in anticancer activity, adapted from Ahmad [13].

Table 1 summarizes the information on the biochemical composition of the fungus, as noted by multiple researchers. The content analysis of the bioactive compound classes includes data on moisture, ash, water-soluble protein content, total lipid content, total carbohydrate content, and dietary fiber content. From Bangladesh, data were reported by Rahman et al. (2020), El Sheikha (2022), and Roy et al. (2018) [21,30,71]. The interest in the nutritional potential of *G. lucidum* in Taiwan was proven by studies conducted by Mau et al. (2001) [70]. The total fatty acid content was reported only by Fraile-Fabero et al. for G. lingzhi from China and *G. lucidum* from mushroom crops from Madrid, Spain, in 2021 [72]. Comparable data were also reported by Parapelli et al. (2021) for *G. lucidum* from India and by Ogbe et al. (2013) from Nigeria [73,74]. Additional studies were published by Wachtel-Galor et al. (2011), by Paterson et al. (2006), and by Garuba et al. (2000) [75,76,77]. The pharmacognostic review of the active compounds isolated from basidiocarp and mycelium of *G. lucidum* revealed that it contains polysaccharides, triterpenes, vitamins, minerals, sterols, proteins, proteo-polysaccharides, lectins, nucleotides, and fatty acids, as reported by Ahmad (2018) [13]. Figure 3 systematizes the categories of biochemical compounds that support the biological activities occurring in the treatment of various cancer tumors [13].

### 3.2. Polysaccharide Content of G. lucidum

Numerous studies have identified the existence of several types of polysaccharides in the fruiting body, spores, or mycelium of *G. lucidum*, as reported by Liu et al. and Lin et al. [78,79]. Polysaccharides represent a class of macromolecules with diverse structures and a wide range of physicochemical properties and biological actions, according to studies by Ferreira et al., Yu et al., and Giavasis [80,81,82]. According to Ahmad, Bhat et al., and Liu et al., these polysaccharide compounds are considered some of the most potent bioactive metabolites with antitumoral effects due to their biochemical structure [13,18,78]. Polysaccharide compounds (Gl-Ps) have a rich history and have been extensively studied in recent years by Sanodiya et al., Parepalli et al., and Chen et al. [27,62,83].

#### 3.2.1. Extraction and Purification of Polysaccharides

**Figure 4:**
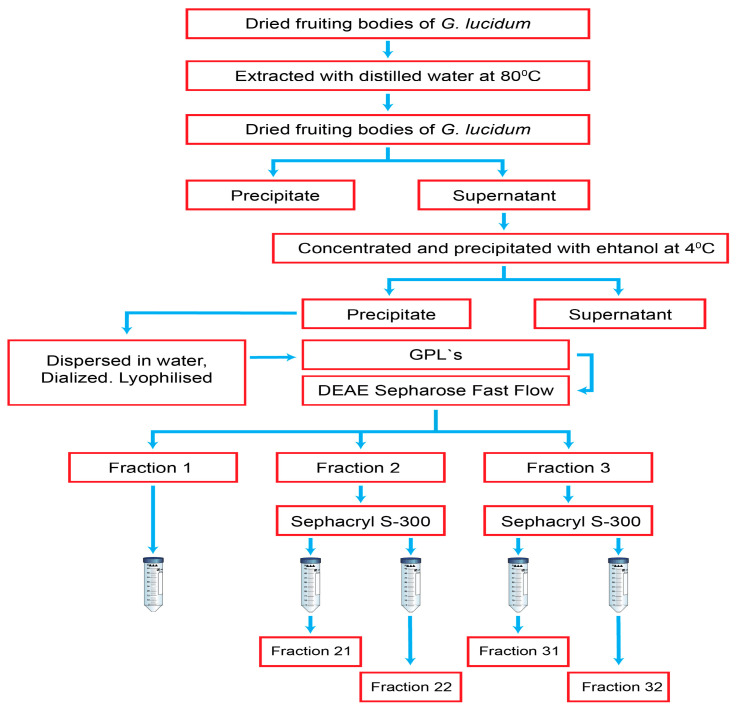
Diagram of the extraction of polysaccharides from *G. lucidum*, adapted from [85].

The most widely used method for polysaccharide extraction from spores, fruiting bodies, and mycelium is hot-water extraction, as documented by Nie et al. [84]. Figure 4 illustrates the extraction of five polysaccharide fractions from *G. lucidum* [85]. The fruiting body of *G. lucidum* was initially peeled, shredded, and sieved in order to obtain a fine powder. The extraction of Gl-Ps from the mushroom powder was performed with distilled water at 80 °C [85]. There are researchers who have used diluted saline solution or alkaline acid solutions for polysaccharide extraction, as reported by Wang et al. (2011). Other extraction methods that were applied included microwaves, ultrasound, or enzymatic methods, as in the studies of Lin et al. (2005), Huang et al. (2010), Zhao et al. (2010), and Leong et al. (2021) [86,87,88,89,90]. After filtering, the solution is subjected to precipitation with alcohol or acetone to obtain crude polysaccharides [84]. Polysaccharide purification can be achieved by several techniques, such as ethanol fractionation or different column chromatographic techniques, as illustrated by Chen et al. (2008), Huang et al. (2011), and Jiang et al. (2012) [91,92,93]. Ion-exchange chromatography (DEAE-Sepharose Fast Flow), gel filtration, and affinity chromatography were the most useful methods, as highlighted by Choong et al. [85]. Figure 4 illustrates the extraction and fractionation steps of polysaccharides from *G. lucidum*. Five polysaccharide fractions were obtained [85].

#### 3.2.2. Structure of Polysaccharides from *G. lucidum*

The investigation of the structures and chemical properties of polysaccharides from *G. lucidum* involves knowledge of the composition of existing monosaccharides, branching structures, types of glycosidic linkages, chain conformations, and molecular weights [18]. As investigation techniques have developed, it was found that *G. lucidum* can have polysaccharides in its composition, either as pure or linked with other proteins or peptides [94,95,96,97,98]. Bhat et al. (2021) showed that homo-glucans from *G. lucidum* are linear or branched biopolymers, possessing a backbone formed by α- or β-linked (1→3), (1→6)-β-glucan and (1→3)-α-glucan glucose units, and may possess side chains attached in different positions [18]. Further investigations by different authors explore bioactivities as well. Bao et al. (2002) determined that the structures of the backbone chains are linear or branched bipolymers [94]. These possess a backbone consisting of α- or β-linked glucose units [94]. In 2006, new structures with heteroglucans were outlined by Cao et al., and new structures with heteropolysaccharides were documented by Sullivan et al. [95,96]. Further types of ligands were presented by Li et al. (2007), Ye et al. (2008), Ye et al. (2009), Wang et al. (2009), Ye et al. (2010), and Liu et al. (2010) [97,98,99,100,101,102]. Pan D. et al. (2012), Ma et al. (2013), and Pan K. et al. (2013) also described the types of polysaccharide structures with different types of linkages, shown in detail in Table 2 [103,104,105]. Ooi et al. (2000) and Zhang et al. (2007) pointed out in their studies that the degree of base-chain substitution and branching chain length play important roles in determining the bioactivity of β-(1→3)-linked glucans [106,107]. Table 2 illustrates various techniques and methods utilized in the extraction, fractionation, and purification of polysaccharides and provides information about their structure (the backbone and monosaccharide compositions).

**Figure 5:**
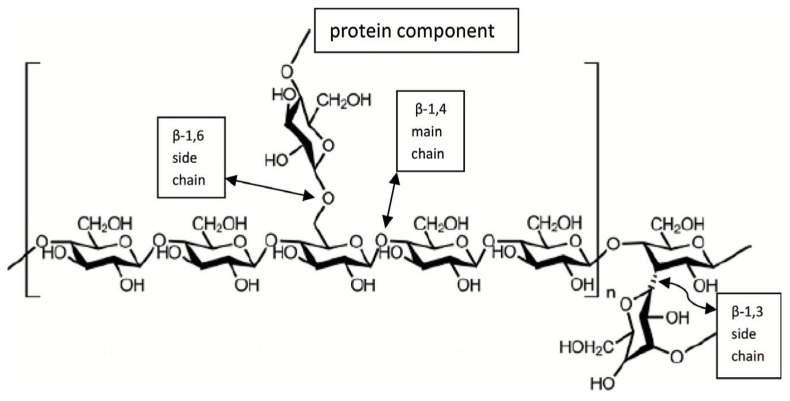
The structure of a Gl-Ps chain consisting of β-(1→3) bonds with some β-(1→6) branches, adapted from [85].

Side-chain branching occurs at C-6 of the main-chain glucosyl residues, as shown in Figure 5 and indicated by Choong et al. (2019) [85]. Jia et al. (2009) argued that among all homo-glucans, β-glucans are glucose polymers that exist as unbranched (1→3)-β-linked backbones in the form of (1→3)-β-linked backbones [108]. Figure 5 shows a Gl-Ps chain [85].

The molecular weight of Gl-Ps can range from 4 × 10^5^ to 1 × 10^6^ Da. Sanodiya et al. (2009) demonstrated that this has a significant impact on reducing cancer progression [27]. Studies by Moradali et al., Doco et al. (2001), and Hung et al. (2005) described polysaccharide-protein or peptide complexes using modern GC-MS methods [109,110,111]. Wang et al. (2002) and Sanodiya et al. (2009) investigated monosaccharides from the raw extract of *G. lucidum* [24,27]. Furthermore, Wang et al. (2009) and Ye et al. (2010) also provided data on monosaccharides identified in *G. lucidum* [100,101]. Similarly, Dai et al. (2010) and Yang et al. (2010) investigated *G. lucidum* and described the monosaccharide composition as predominantly consisting of xylose, fructose, glucose, and maltose [112,113]. Several other studies have been published reporting data on the polysaccharide structure and their medical applications, including Dong et al. (2012), Liu et al. (2012), Skalicka-Wozniak et al. (2012), and Pascale et al. (2022) [114,115,116,117].

### 3.3. Triterpene and Triterpenoid Content of G. lucidum

Triterpenes belong to the class of terpenes that have a molecule consisting of six isoprene units and are widespread in the plant kingdom [35]. Triterpenoids are part of the triterpene class, having heteroatoms (usually oxygen atoms) in the molecule. They are important bioactive compounds in the composition of *G. lucidum*, as shown by Wu et al. (2023) [118]. In 2018, Gu et al. revealed that the present triterpenes synthesize structures derived from lanosterol, which has a skeleton with a tetracyclic structure and the molecular formula C_30_H_48_ [119]. Terpenoid compounds from *G. lucidum* that have a C30 backbone and molecular masses between 400 and 600 kDa were studied by Baby et al. and Galappaththi et al., who generically named them Ganoderma triterpenoids (Gl-Ts) [120,121]. Further research on the structure and characterization of triterpenoids from *G. lucidum* that elucidates their biological activities was published in 2022 by Cör et al. and Lin et al. [122,123].

#### 3.3.1. Physicochemical Determination and Analysis of Triterpene Compounds

Studies conducted by Ghorai et al. (2012), Taofiq et al. (2017), and Chang et al. (2012) utilizing UV spectrophotometric measurements have made significant contributions to the methods for determining total terpenoids [124,125,126]. Huie et al. (2004) employed chromatographic and electrophoretic methods for the analysis of triterpenoids, and Yang et al. (2007) utilized a combined approach involving HPLC-ESI-MS [127,128]. Chen et al. (2012) highlighted the existence of multiple methods for investigating terpenoids [83]. Triterpenoid analysis was also outlined in the studies of Zhang et al. (2008), Shi et al. (2010), and Hadda et al. (2015) [129,130,131]. Che et al., Hui et al., and Zhang et al. reported novel insights into *triterpenoids* obtained from *G. lucidum* [132,133,134]. In 2023, Wu et al. emphasized the necessity of a new research strategy intertwining the concepts of chemical component analysis and pharmacological activity [118].

#### 3.3.2. Structure of *G. lucidum* Triterpenoids (Gl-Ts)

**Figure 6:**
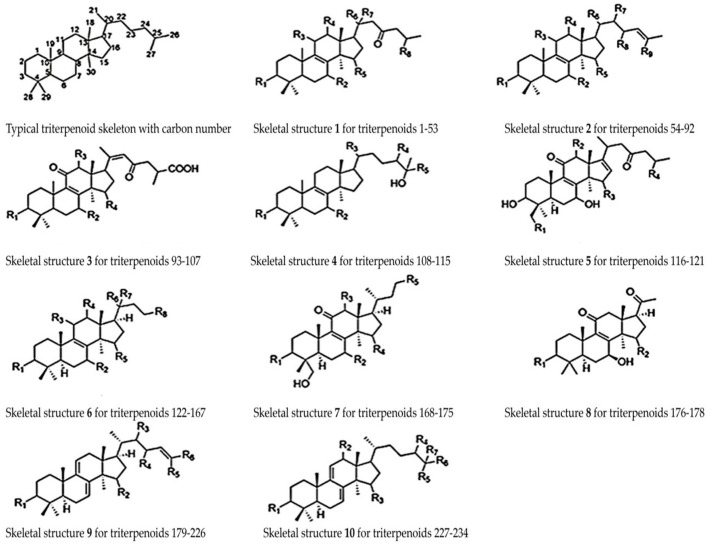
Skeletal structure of a typical terpenoid and other terpenoids 1–10 [118].

Wu et al. (2023) extensively reported on the diverse types of Gl-Ts identified from the mycelia, fruit body, and spores of *G. lucidum*, showcasing distinct structural variations, including alcohols, aldehydes, ketones, acids, esters, and various other substituents positioned differently [118]. Xia et al. (2014) conducted an in-depth analysis of the skeletal structure of *G. lucidum*, revealing that a majority of these terpenoids consist of 30 carbon atoms [135]. Using information derived from Wu et al., in Figure 6, the structures of a typical terpenoid skeleton are depicted, illustrating the numbered positions corresponding to the matched carbon atoms alongside 10 additional terpenoid structures exhibiting diverse substituents [118].

**Figure 7:**
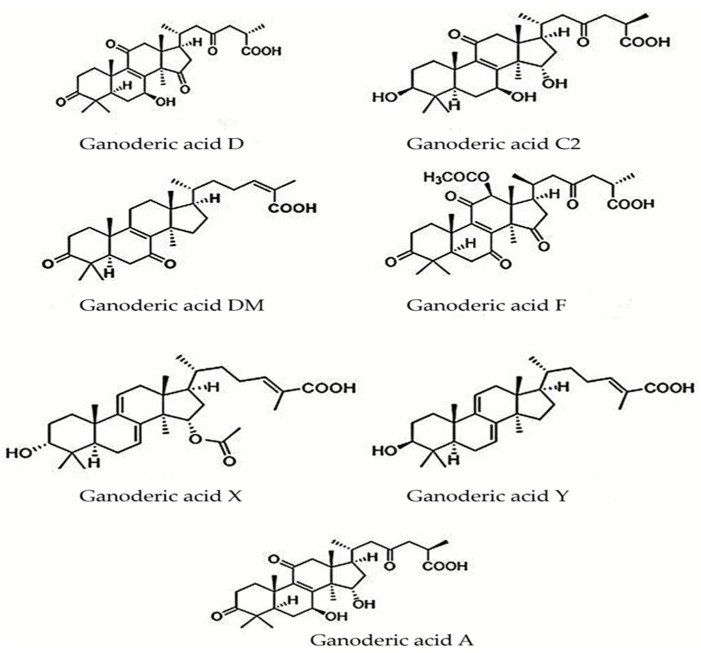
Structure of ganoderic acids from *G. lucidum*, adapted from [122].

In Figure 6, illustrating the typical terpenoid skeleton structure, distinct substituents are evident at positions C-3, C-7, C-11, C-12, C-15, C-20, and C-27. Fatmawati et al. (2010) elucidated the structure of a new terpenoid, Ganoderic acid Df, featuring a β-hydroxy substituent at the C-11 position, distinguishing it from all other compounds characterized by a carbonyl group at the same position [136]. At the C-3 position, potential substituents include the β-hydroxy, carbonyl, and β-acetoxy groups. At C-20, a single carbon atom can host two substituents, which may be the methyl or hydroxyl groups or even hydrogen. Wu et al. demonstrated that at the C-25 position, various carboxyl groups, such as formyl, acetyl, or butyryl, can be found [118]. Additionally, Sharma et al. (2019) presented data on triterpenes from *G. lucidum*, characterized by isoprene units in their composition, featuring a C30 skeleton structure of ganoderic acids, aldehydes, esters, alcohols, lactones, glycosides, ketones, and molecular masses ranging from 400 to 600 g/mol [137]. Koo et al. (2019) identified a new compound with a lanostane triterpenoid structure named Ganosidone A, which, along with eight other derivatives, was investigated for its cancer chemopreventive potential [138]. Concerning the molecular configuration of ganoderic acids, Cör et al. (2022) documented the structures illustrated in Figure 7 [122].

**Figure 8:**
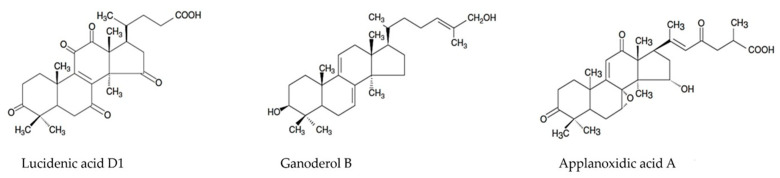
Specific structures for other ganoderic compounds [152].

Murata et al. (2019) identified and extracted a novel compound with a lanostane triterpene structure from *G. lucidum* using NMR and MS physicochemical analysis [139]. Studies presented by Yue et al. in 2008 and 2010, as well as those by Zhang et al. and Cheng et al., revealed the cytotoxic effects exerted by ganoderic acids from *G. lucidum*, which alter proteins involved in cell proliferation and cell death in carcinogenesis or oxidative stress [140,141,142,143]. In 2012, Liu et al. and Rios et al. studied the lanostanoid compounds from *G. lucidum* for their anticancer activities [144,145]. In 2013, Li et al. isolated a novel ganoderic acid from *G. lucidum* mycelia and studied its characteristics. Fatmawati et al. studied the structure–activity relationship of lanostane-type triterpenoids, and Li et al. reported cytotoxic effects in a wide range of triterpene compounds [146,147,148]. In 2017, Chen et al. presented data on compounds with triterpene structures existing in *G. lucidum* that exhibited activity as inhibitors of biological processes [149]. In 2019, Liang et al. illustrated their findings on the structure and mechanisms of action of ganoderic acids from *G. lucidum* [150]. In 2021, Chinthanom et al. documented their findings on lanostane triterpenoids isolated from mycelial cultures of Ganoderma spp. that can be modified by semisynthesis, thus obtaining synthetic compounds [151]. In 2023, Pascale et al. reported data on the specific structures and pharmacological mechanisms of triterpenoids with biological activities from *G. lucidum* [152]. Figure 8 illustrates other structures specific to the terpenoid compounds described by Pascale et al. [152].

### 3.4. Vitamins, Minerals, and Sterols Content

Vitamins, minerals, and sterols were among the 400 biologically active constituents recognized by various researchers after 2018, including Cör et al., Yang et al., and Ahmad, F. In terms of quantity, the vitamins were in the following order: B1, B2, B6, β-carotene, C, D, and E [153,154,155]. Hussein et al. (2022) also studied the existence of vitamins in *G. lucidum* [156]. El Sheikha et al. (2022) highlighted the highest vitamin content as consisting of niacin (B3) and ascorbic acid (C) [30]. Mineral contents have been reported by several studies, such as El Sheikha et al., Roy et al., Ogbe et al., Cör et al., and Hussein et al. [30,71,74,153,156]. Table 3 displays the values for mushroom compositions reported by various authors, expressed in milligrams per 100 g (mg/100 g), parts per million (ppm), and percentages (%) [30,71,74,153,156].

Similar data were also presented by Sharif et al. (2016) and Treviño et al. [157,158]. The bioaccumulation of copper and zinc in *G. lucidum* was also documented by Matute et al. in 2011 [159]. In 2008, Falandysz J. reported the existence of selenium in the composition of some antioxidant enzymes with protective actions that are involved in antitumor effects [160]. In their study, conducted in 2000, Chiu et al. identified the presence of germanium in the Ganoderma extract. Their research encompassed an assessment of genotoxicity and antigenotoxicity related to this finding [161]. Du et al. (2008) studied the positive effect of selenium on the immune regulation activity of *G. lucidum*, and the involvement of this element in anticancer activities was documented [162].

Sterols with the chemical formula C_17_H_28_O are a group of steroids that are cyclic secondary monohydric alcohols. Since 2002, Ma et al. have reported three new lanostanoids and two ergostane sterols, which were isolated by spectroscopic methods [163]. The role of sterols is important in the body’s metabolism in regulating some hormonal and immune system functions. Akihisa et al. (2007) studied the effects of sterols and triterpene acids from *G. lucidum* on anti-inflammatory and antitumor actions [164]. In 2011, the sterol content of *G. lucidum* was researched by Liu et al., who analyzed it qualitatively and quantitatively using HPLC methods [165]. In 2015, Baby et al. showed that sterols in *G. lucidum* can be classified based on their skeletons [120].

### 3.5. Protein, Lectin, and Amino Acid Content

#### 3.5.1. Proteins and Peptides in *G. lucidum*

Proteins constitute a distinct category of biochemically active molecules in G. *lucidum*, as demonstrated by Cör et al. in 2018 [153]. Numerous studies have reported various biological effects of the primary fungal protein, Ling-zhi-8 (LZ-8). Structural reports were published by Huang et al. in 2009, and the immunomodulatory actions of LZ-8 were documented by Hsu et al. in 2013, Lin et al. in 2014, and Yang et al. [166,167,168,169]. In 2015, Sa-ard et al. studied crude proteins from *G. lucidum* (both mycelia and fruiting bodies) for their antioxidant actions [170]. In 2021, Fraile-Fabero et al. conducted studies on proteins from *G. lucidum* [72]. Additional research by Sun et al. in 2004 revealed the existence of compounds with a polysaccharide-peptide complex structure along with phenolic components with antioxidant potential in *G. lucidum* [171].

Ji et al. (2007) further documented the existence of proteo-polysaccharides in *Ganoderma lucidum*. They studied the immunomodulatory effects, emphasizing the anticancer properties of this fungus [172]. The immunomodulatory properties of LZ-8 protein and polysaccharides were studied by Yeh et al. in 2010 and Girjal et al. in 2012 [173,174]. Zhong et al. (2015) conducted research on the involvement of the peptide-polysaccharide complex from *G. lucidum* in oxidative stress [175]. Subsequent data on the presence of proteins in *G. lucidum* and their biological actions were published by Kumakura et al. in 2019, and further insights were provided by Yu et al. in 2021 and 2023, where they specifically documented the effects of proteoglycans derived from *G. lucidum* [176,177,178]. Huang et al. (2022) reported a novel pentapeptide in the mycelium of Ganoderma spp. that demonstrates antioxidant properties [179].

#### 3.5.2. Lectins from *G. lucidum*

Lectins are glycoproteins found in the fruiting body of *G. lucidum*. Lectins are carbohydrate-binding proteins that have been classified according to their origin and structure. In 2007, Thakur et al. isolated and purified a group of lectin-structured proteins with a weight of 114 kDa from the fruiting bodies of *G. lucidum* [180]. In 2011, Girjal et al. isolated a new bioactive lectin from *G. lucidum*, which also exhibited hemagglutinating activity against both human and animal erythrocytes [181]. Nikitina et al. (2017) investigated lectins in mycelia and detected hemagglutinating activity in culture medium and in a crude mycelial extract of *G. lucidum* [182]. Recent research conducted by Yousra et al. has demonstrated varying binding patterns of lectins from *G. lucidum* with glycan moieties. These patterns play a crucial role in influencing biological activities, including antitumor, antiviral, and immunomodulatory effects [183].

#### 3.5.3. Amino Acid Content of *G. lucidum*

The amino acid content has been documented by several authors. According to the research of Sanodiya et al., Yousra et al., and Deepalakshmi et al., the most abundant amino acid was glutamic acid [27,183,184]. This was followed by aspartic acid, glycine, and alanine. The amino acids in *G. lucidum* have also been analyzed by Zhang et al. in 2018, who reported 18 different types of amino acids [185]. It should be noted that factors such as the origin of the tested samples and the species of fungus, as well as different analysis techniques, may result in different amino acid values. Compounds with antioxidant properties from *G. lucidum* have been analyzed in numerous studies. Thus, Kim et al. (2008), Sheikh et al. (2014), Lin et al. (2015), and Veljović et al. (2017) presented data on the chemical compounds of *G. lucidum* [186,187,188,189]. The amino acid values for *G. lucidum*, as reported by Sanodiya et al. in 2009 and Deepalakshmi et al. in 2011, are presented in Table 4 [27,184].

#### 3.5.4. Content of Compounds with Antioxidant Properties in *G. lucidum*

Dong et al. (2019), Zheng et al. (2020), Rahman et al. (2020), and Kolniak-Ostek et al. (2022) reported data regarding the total triterpenoid, polysaccharide, polyphenol (TPC), and flavonoid (TFC) content of *G. lucidum* [21,190,191,192]. Furthermore, Kim et al. (2008) analyzed the phenolic compounds from *G. lucidum* using HPLC and identified 28 phenolic compounds. All these compounds are responsible for antioxidant activity [186]. Triterpenoid compounds were documented by Lin et al. in 2015 and Kolniak-Ostek et al. in 2022 [188,192]. Polysaccharide compounds were described by Lin et al. in 2015 [188]. Polyphenolic compounds were investigated by Rahman et al. in 2020 and by Kolniak-Ostek et al. in 2022 [21,192]. Flavonoid compounds and ascorbic acid were detailed in a study by Rahman et al. in 2020 [21]. Depending on the area of origin of the mushroom (cultivated or forest) and the extraction techniques used, the concentrations of the compounds listed in Table 5 vary considerably. The terpenoid content is lower in cultivated *G. lucidum*, depending on the cultivation method, as Kolniak-Ostek et al. showed [192]. The results on compounds responsible for antioxidant activity are presented in Table 5.

Kolniak-Ostek et al. presented more data on several phenolic acids present in *G. lucidum* responsible for antioxidant activity, which are presented in Table 6 [192].

The antioxidant properties of G. *lucidum* were analyzed using several methods, including the reporting of DPPH and ABTS radical scavenging activities, as well as the FRAP assay. The results were expressed in units of measurement. These units vary according to the different experimental methods used. However, the importance of the results lies in the unequivocal demonstration of the antioxidant activity possessed by the constituents found in *G. lucidum* (see Table 7).

Furthermore, Dong et al. (2019) demonstrated a strong correlation between the antioxidant capacities measured using DPPH, ABTS, and FRAP assays and the content of polyphenolics and triterpenoids in *G. lucidum* [190]. Similar studies have also been published by Saltarelli et al. in 2015 [193]. The properties of compounds in *G. lucidum* and their effects on antioxidant activity were also documented by Tang et al. (2016), Sanchez C. (2017), Mohammadifar et al. (2020), and Mustafin et al. (2022) [194,195,196,197].

### 3.6. Content in Nucleosides and Fatty Acids

#### 3.6.1. Nucleosides and Nucleobases in *G. lucidum*

Nucleotides are monomeric units that consist of a base (purine or pyrimidine) and one or more phosphate groups. Nucleotides are formed from nucleosides by phosphorylation under the action of kinases. Nucleosides are glycosyl amines derived from a nitrogenous base and a ribose or deoxyribose. These compounds have been shown to contain uridine and uracil, both of which are capable of reducing elevated serum aldolase levels. The water-soluble fraction of *Ganoderma* suppresses platelet aggregation. Cheung et al. have carried out studies on the identification and role of bases and nucleoside markers [198]. Gao et al. (2007) identified six nucleobases qualitatively in fruiting body samples of *G. lucidum* and *G. sinense* [199]. Additional studies of the distribution of nucleotides and nucleobases were also carried out by Yuan et al. in 2008 and Chen et al. in 2012, who were able to document 16 nucleotides and nucleobases in the *Ganoderma* species [200,201]. Phan et al. (2018) demonstrated in their work that nucleobases, nucleosides, and nucleotides found in fungi play crucial roles in regulating various physiological processes in the human body through purinergic and/or pyrimidine receptors. [202]. In 2022, Sheng et al. analyzed 10 target compounds from 23 batches of *Ganoderma* samples from different regions of China and demonstrated that the geographical origin of the fungi might be the exclusive factor affecting the accumulation of nucleosides and nucleobases in the *Ganoderma* spp. mycelium [203].

#### 3.6.2. Fatty Acid Content

The total lipid content of *G. lucidum* has been reported in several studies by Rahman et al. (2020), El Sheikha (2022), Roy (2018), and Ogbe et al. (2013) [21,30,71,74]. Comparative data for the fatty acid content of carpophores from G. lingzhi (from industrial cultures in China) and *G. lucidum* (from cultures in Madrid, Spain) were described by Fraile-Fabero et al. in 2021 [72]. Fraile-Fabero et al. found that G. lingzhi contains the highest percentage of arachidic acid, followed by the margaric, behenic, margaroleic, lignoceric, and cis-vaccenic acids, while *G. lucidum* contains the highest percentage of α-linolenic acid, followed by the myristic, stearic, capric, erucic, nervonic, elaidic, octadecatrienoic, octadecadienoic, and eicosatrienoic acids [72]. Data on the presence of essential fatty acids in *G. lucidum* were also presented by Hossain et al. in 2007 [204]. Stojković et al. (2014) conducted comparative studies between the *G. lucidum* species from Serbia and China [205]. Lin et al. (2017) reported hydroxy fatty acids (HFA) present in the molecular species of acylglycerols of *G. lucidum* [206]. Salvatore et al. (2020) identified fatty acid methyl esters (FAMEs) in the triglycerides of *G. lucidum* spores [207]. Table 8 displays the composition similarities between the two species. [72].

Phytochemical reports over the past four decades indicate the presence of 279 bioactive secondary metabolites (Wasser et al.), along with over 200 polysaccharides and polysaccharide-protein complexes (Baby et al.), and more than 30 steroidal compounds (Wasser et al.) within the chemical composition of *G. lucidum*. These compounds play a pivotal role in the development of various biological actions. [120,208,209].

## 4. Biological Activities against Cancer

The biocomponents present in *G. lucidum* contribute to the numerous biological actions of this medicinal mushroom, making it applicable in medical contexts for combating cancer. Both polysaccharide compounds (Gl-Ps) and triterpene compounds (Gl-Ts) have been shown to exhibit anticancer activities. Over a 25-year period, numerous studies have investigated the anticancer effects of *G. lucidum* compounds, including those reported by Wang et al. (2002), Zhang et al. (2007), and Akihisa et al. (2007) [24,107,164]. Between 2009 and 2015, other researchers published studies on the anticancer effects of *G. lucidum* bio-compounds, such as Cho et al., Ferreira et al., Trajkovic et al., Kao et al., Zhao et al., and Joseph et al. [15,28,34,35,37,80,89]. Between 2018 and 2023, new studies disclosed data on anticancer actions, including those by Ahmad F. and Cör et al. in 2018, Kolniac-Ostek et al. in 2022, and Ekiz et al. in 2023 [13,153,192,210]. Our aim is to review the most important studies and systematize these data on the possible mechanisms.

### 4.1. Anticancer Action

The anticancer activity attributed to Gl-Ps was studied by Cao et al. in 2002 and 2003 [211,212]. Many other studies have demonstrated the effect of Gl-Ps on cytokines, including those by Chen et al. and Zhu et al. [213,214]. In 2007, Zhu et al. demonstrated the beneficial immunological effects in mice attributed to Gl-Ps extracts administered at low doses [215]. You et al. studied the effects of Gl-Ps on antioxidant enzymes in ovarian cancer in rats, while Xu et al. conducted research on mediating the immunomodulatory, cytotoxic, and anti-angiogenic effects of Gl-Ps [216,217]. Polysaccharides have been identified as a distinct class of compounds present in *G. lucidum* compositions, demonstrating anticancer activity by stimulating host immune function, as reported by Ahmad F., Sun et al., and Wiater et al. [213,218,219]. In 2012, Zhang et al. reported the effects of Gl-Ps on suppressing hepatocyte proliferation in rats [220]. Pan et al. reported the effects of Gl-Ps on rats with gastric cancer, inducing enhanced immunity and antioxidant activity [103]. The study by Suarez-Arroyo et al. in 2013 explored inflammatory breast cancer (IBC) and the effects of Gl-Ps in breast cancer [221]. In 2015, Habijanic et al. reported on the effects of Gl-Ps in modulating cytokine responses and lymphocyte activity [222]. Several studies have delved into the mechanisms of action of *G. lucidum* compounds in cancer treatment, such as those published by Ahmad F., Sohretoglu et al., and Wang et al. in 2018, as well as Fu et al. in 2019 [13,223,224,225]. The triterpene compounds (Gl-Ts) identified in *G. lucidum* exhibit significant anticancer effects. Both Min et al. in 2000 and Gao et al. in 2002 reported that triterpene compounds displayed cytotoxic effects against tumor cells [226,227]. In 2004, Lin et al. investigated the actions of Gl-Ps from aqueous extracts and Gl-Ts from alcoholic extracts of *G. lucidum*, evaluating their angiogenic effects [228]. Li et al. conducted research on ganoderic acid X, which acts by inhibiting topoisomerases and inducing apoptosis [229]. In Table 9, we have compiled the effects of essential compounds (Gl-Ps and Gl-Ts) in *G. lucidum* that significantly contribute to its anticarcinogenic activity.

Tang et al. (2006) reported the effects of ganoderic acid T in inhibiting lung cancer tumors (95-D), inducing apoptosis, and arresting the cell cycle at the G(1) phase [230]. Gl-Ts-type triterpenes and ganoderic acids A, F, and G were investigated in 2008 by Jiang et al. for their effects on breast cancer [231]. In 2009, Trajkovic et al. documented the action of triterpenes in B-16 melanoma, while Xu et al. studied ganoderic acid T and its effects on lung cancer [34,232]. In 2017, Barbieri et al. published data on the inhibition of IL-8, IL-6, MMP-2, and MMP-9 release in cancer cells under pro-inflammatory conditions in breast cancer and melanoma cells, and Ye et al. disclosed data on the effects of ganoderic and lucidenic acids in inhibiting the growth of lung carcinoma metastases and suppressing colon inflammation [233,234]. In 2020, the anticancer effects of triterpene fractions extracted from *G. lucidum* on SW620 human colorectal cancer cells were reported in vitro by Li et al. [235]. The action of Gl-Ts against various cancers has been studied by Jiao et al. (breast cancer cells), Wang et al. (prostate cancer), and Liu et al. (colorectal cancer) [236,237,238]. In 2022, Shahid et al. conducted research on the anticancer activities of Gl-Ts in skin cancer [239].

### 4.2. Possible Mechanisms in Anticancer Actions

The medical world has shown significant interest in establishing possible mechanisms of anticancer activity, especially as the literature provides ample evidence regarding the actions of *G. lucidum* biocomponents against various forms of cancer. In 2018, Ahmad F. systematized the possible mechanisms by which *G. lucidum* bio-compounds participate in cancer treatment [13]. The steps considered by Ahmad include the activation of the host cell immune response, induction of cell differentiation, inhibition of angiogenesis, direct cytotoxicity to tumor cells, inhibition of urokinase-type plasminogen activator and receptor expression in cancer cells, and inhibition of phase II metabolizing enzymes [13].

**Figure 9:**
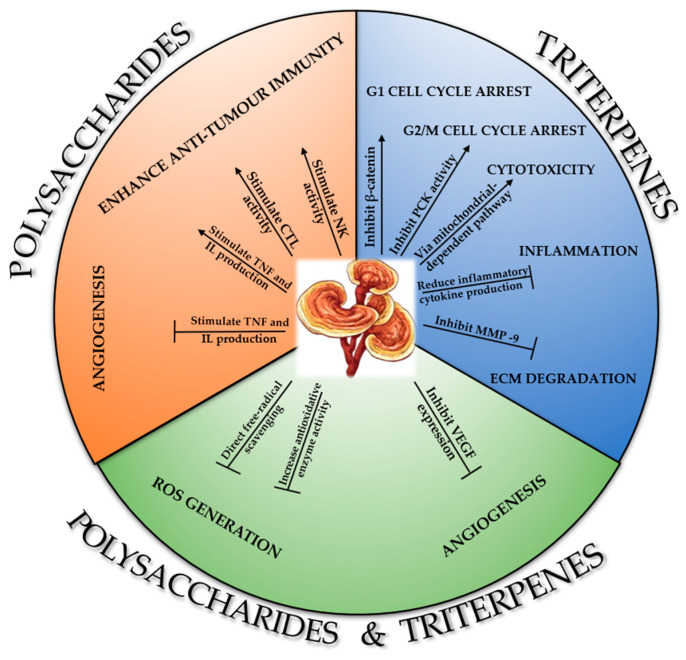
Mechanisms of action of polysaccharide and triterpene extracts isolated from *G. lucidum* with anticancer effect, adapted from [35].

Kao et al. elucidated the mechanisms of the anticancer action of *G. lucidum* compounds by discussing the distinct effects of polysaccharide and triterpene compounds separately, as well as their combined effects (see Figure 9) [35].

They showed that polysaccharide compounds (Gl-Ps) act through three mechanisms [35]: by enhancing the host immune response, by stimulating macrophage activity, and by stimulating T-lymphocytes and natural killer (NK) cells. Triterpenes (Gl-Ts) act through five mechanisms:

- G1-phase cell cycle arrest by inhibition of β-catenin;
- Inhibition of protein kinase C (PCK), which generates G2-phase cell cycle inhibition;
- Induction of apoptosis in cancer cells via the mitochondrial pathway, followed by activation of caspase cascades;
- Preventing tumor metastasis by inhibiting MMP-9 and interleukin IL-8 and by degrading the extracellular matrix (ECM);
- Suppressing the secretion of anti-inflammatory cytokines [240].

Together, the two major classes of bio-compounds, Gl-Ps and Gl-Ts, share two common mechanisms, namely:

I. Antioxidant actions by reducing oxidative stress generated by free radicals and reactive oxygen species (ROS) through the actions of antioxidant enzymes [35].
II. Suppressing angiogenesis and inhibiting nitric oxide production.

### 4.3. Other Biological Actions Involved in Anticancer Activities

The antitumor activity of *G. lucidum* bio-compounds is based on the differential effects of other biological activities generated by *G. lucidum* bio-compounds, as reported by Cör et al. [122,153]. Other researchers, such as Sharma et al., Ahmad, and You et al., have also conducted studies on the various factors that can lead to cancer [137,155,216]. Additionally, researchers like Xu et al., Fu et al., and Hapuarachchi et al. have analyzed various biological actions associated with cancer [217,225,241].

#### 4.3.1. Immunomodulatory Activities

Anticancer activity based on immunomodulation activity has been reported by several researchers, such as Bao et al., Ooi et al., Moradali et al., and Lin et al. [94,106,109,168].

##### Effects of Gl-Ps on T- and B-Lymphocytes

Numerous studies have been reported that highlight the activating role of Gl-Ps (F3 fraction) on T-lymphocytes by increasing interleukin production: IL-1, IL-2, IL-6, and IL-12, and increasing the expression of IFN-γ and INF-α. Additionally, they enhanced DNA synthesis in mouse spleen cells, as reported by Chen et al. [213] and Wang et al. [24]. Gl-Ps from *G. lucidum* can activate PKC and PKA protein kinases in murine T-lymphocytes, according to Sohretoglu et al. [223]. The effect of Gl-Ps on B-lymphocytes is also crucial for tumor immunity. For example, Gl-Ps can activate B-lymphocytes by enhancing their proliferation and differentiation, leading to the production of substantial amounts of immunoglobulins in mice. Furthermore, Gl-Ps can directly stimulate the expression of PKCα and PKCγ in B-lymphocytes, as reported by Zhang et al. [242]. Activated B-lymphocytes increase the production of immunomodulatory substances, such as IL-1β and TNF-α, and reactive nitrogen species, such as NO [242].

##### Effect of Gl-Ps on Dendritic Cells

Dendritic cells (DCs) are professional antigen-presenting cells involved in the initiation of the primary T-lymphocyte immune response [223]. Stimulation of dendritic cell maturation by Gl-Ps from *G. lucidum* was documented by Sanodiya et al. [27]. Lai et al. reported that treatment with Gl-Ps F3 fraction extract improved the mixed lymphocyte response and stimulated the production of ten cytokines and six chemokines [243]. Jan et al. outlined the immunomodulatory activity of Gl-Ps, including the activation and maturation of DCs, as evidenced by increased cytokine production (IL-12, IL-6, IL-23, and IL-10) [244]. Chan et al. reported that treatment of monocytic leukemic cell lines with Gl-Ps resulted in increased leukocyte HLA-DR antigen expression and induced leukemic cell differentiation through increased superoxide production and cell cycle arrest [245].

##### Effect of Gl-Ps on Macrophages

Sohretoglu et al. demonstrated that Gl-Ps activate macrophages in vitro and elevate the levels of various cytokines, including IL-1β, tumor necrosis factor (TNF-α), IFN-γ, and IL-6 in the culture medium. This activation significantly increases macrophage phagocytosis and enhances macrophage-mediated tumor cytotoxicity [223]. Zhang et al. showed that the in vivo treatment of S180 sarcoma-bearing mice with Gl-Ps activated bone marrow-derived macrophages, inducing the production of immunomodulatory compounds such as IL-1β, TNF-α, and nitric oxide (NO) [242]. Hsu et al. elucidated the role of caspases in macrophage F3 fraction-induced Gl-Ps from *G. lucidum* spores [246]. Guo et al. discovered a novel water-soluble polysaccharide within *G. lucidum* spores that acts as an inducer of TNF-α and IL-6 secretion in murine peritoneal macrophages [247]. The in vivo administration of extracts from *G. lucidum* spores potentiated the proliferative response of splenocytes and induced antitumor activity against lung cancer in mice [247]. Hsu et al. reported that Gl-Ps induces increased secretion of the inflammatory cytokine IL-1 and stimulates the expression of pro-IL-1 and IL-1-converting enzymes in human and murine macrophages, an association linked with its anticancer activity [248].

##### Effect of Gl-Ps on Natural Killer (NK) Cells

Altfeld et al. defined the role of (NK) natural killer cells in innate immunity [249]. Chien et al. demonstrated that treatment with Gl-Ps resulted in increased monocyte, macrophage, and NK cell populations in human umbilical cord blood [250]. Wang et al. studied the effects of the bio-compounds from *G. lucidum* that, upon oral administration to mice, improved NK cell and phagocytosis activities and increased cytokine levels [251]. In additional research by Zhu et al., it was shown that Gl-Ps accelerated the recovery of bone marrow cells, red blood cells, and white blood cells, as well as splenic NK and NKT killer cells, and enhanced T- and B-lymphocyte proliferative responses [215]. The application of Gl-Ps treatments is recommended in cancer chemotherapy only at low doses [215].

#### 4.3.2. Anti-Proliferative, Cytotoxic, and Apoptosis-Increasing Activities

Ganoderic bio-compounds have demonstrated various anti-proliferative and cytotoxic effects in studies regarding the treatment of different types of cancer [252,253,254,255]. The anti-proliferative effects of Gl-Ts compounds in *G. lucidum* manifest through cell cycle arrest [252,253]. Gl-Ts compounds can arrest the cell cycle in the G1 phase by inhibiting the β-catenin pathway, as reported by Wu et al., and in the G2/M phase by suppressing protein kinase C (PKC) activity, as reported by Lin et al. [252,253]. Jedinak et al. reported that ganodermanontriol inhibited the proliferation of HCT116 and HT-29 colon cancer cells by inhibiting β-catenin [254]. Li et al. (2005) identified ganoderic acid X as a compound that can arrest the cell cycle by inhibiting topoisomerase [229]. Chen et al., in 2010, reported that ganoderic acid T (GA-T) exhibits anti-proliferative effects against cancer cells in vitro and against metastasis in vivo [255]. In 2008, Chen et al. reported in the wound vacuolization assay that ganoderic acid Me (GA-Me), administered in a dose- and time-dependent manner, inhibited tumor invasion and cell adhesion to the extracellular matrix (ECM) [256]. GA-Me suppressed master metalloproteinases at the mRNA and protein levels in 95-D cells and is considered a potent antimetastatic carcinoma inhibitor [256]. Hsu et al. studied the anti-proliferative effects of lucidenic acids in human leukemic HL-60 cells [257].

Tang et al. conducted a study on the anti-proliferative effect of ganoderic acid T against cancer cells and observed enhanced cytotoxicity in lung cancer [230]. Triterpene compounds can induce apoptosis of cancer cells via the mitochondria-dependent pathway, followed by caspase activation, as reported by Kao et al. and Liu et al. in 2011 and 2012 [35,258,259]. Zhou et al. investigated the cytotoxic effects of GA-Me in human colon carcinoma, observing a dose-dependent pattern, and determined that the anticancer bioactivity of GA-Me was mediated through induced apoptosis [260].

#### 4.3.3. Anti-Inflammatory Activities

Several inflammatory mediators, such as TNF-α, IL-6, TGF-β, and IL-10, have been shown to play roles in cancer initiation and progression [261,262]. *G. lucidum* biocomplexes have demonstrated dose-dependent anti-inflammatory effects [223]. Administration of the triterpene extract suppressed inflammatory cytokine secretion in macrophages with a significant reduction in inflammation in the affected tissue, as reported by Dudhgaonkar et al. in 2009 [263]. Joseph et al. found that Gl-Ps administration resulted in a 58% inhibition of inflammation, as assessed by carrageenan-induced (acute) and formalin-induced (chronic) inflammation assays [37].

#### 4.3.4. Anti-Angiogenic Activities

Angiogenesis, the process by which new vasculature is formed from pre-existing vasculature, plays a key role in tumor growth and metastasis as well [264]. Nitric oxide is known to be an angiogenesis-inducing agent in tumors, promoting capillary formation within the tumor and allowing it to expand. In 2004, Cao et al. reported the existence of a peptide in *G. lucidum* that significantly reduced microvessel formation, as detected by the chorioallantois membrane assay [265]. Stanley et al. (2005) found that the *G. lucidum* extract prevented capillary morphogenesis by inhibiting the secretion of angiogenic factors VEGF and (TGF)-β1 [38]. Cao et al. (2006) demonstrated that the *G. lucidum* extract contains a polysaccharide peptide that exhibits anti-angiogenic activity by inhibiting nitric oxide production, subsequently suppressing cell multiplication in a dose-dependent manner [95].

#### 4.3.5. Antioxidant Activities

Excess free radicals adversely affect bases in the nucleic acid structure, amino acids in the protein structure, and double bonds in unsaturated fatty acids, leading to oxidative stress, which is responsible for the alteration of DNA, RNA, proteins, and lipids. Hsieh et al. reported that bio-compounds from *G. lucidum* can exert chemopreventive effects through their antioxidant properties, such as free radical scavenging, as well as the ability to affect phase II detoxification enzymes [39]. Other authors, such as Smina et al., studied the antioxidant activity of Gl-Ts and demonstrated that they can reduce free radicals in cancer cells. [266]. In another study, Smina et al. reported the effect of total triterpenes from *G. lucidum* on the intracellular levels of reactive oxygen species (ROS) and the activities of endogenous antioxidant enzymes in spleen lymphocytes, highlighting their role in reducing radiation-induced oxidative DNA damage in spleen cells [267]. In 2001 and 2003, Lu et al. highlighted the beneficial contribution of the polysaccharides extracted from *G. lucidum* mycelium in reducing ROS-induced oxidative damage [268,269]. Lee et al. reported the existence of an amino polysaccharide compound in *G. lucidum* that can inactivate hydroxyl and superoxide anion radicals [270]. Other authors, such as XiaoPing et al. and Zhao et al., studied Gl-Ps and showed that these bio-compounds could be beneficial for glutathione peroxidase and reduce malonaldehyde levels in rats with cervical carcinoma and mice exposed to γ-irradiation [49,271].

## 5. Toxicity and Safety

Although there are numerous published studies on the beneficial effects of *G. lucidum*, there is also relatively little information reporting the toxic effects in humans. In this regard, Ahmad F. highlighted human sensitisation to *Ganoderma* antigen, reported in the USA, leading to allergic reactions [13,155]. When undergoing *G. lucidum* treatment, special attention should be paid to potential interactions with other drugs. Diabetic patients or those being treated with anticoagulants or antiplatelet drugs require special caution when being administered *G. lucidum*, as the anticipated effects may be altered [272].

In cancer therapy, although *G. lucidum* has been utilized as an anticancer agent, caution is still required when using it in conjunction with chemotherapy due to potential toxicity. Plasma concentrations of *G. lucidum* should be carefully monitored to detect elevated, toxic levels [273]. In an *in vitro* study, *G. lucidum* extracts were found to have toxic effects when exposed to cells at concentrations higher than those required for stimulatory results, resulting in a significant reduction in cell viability in a number of cell lines [274]. *G. lucidum* exhibits antihypertensive activity and may potentiate the effects of antihypertensive drugs [275]. Gl-Ps from *G. lucidum* have antibacterial activity and can enhance the activity of some antibiotics (e.g., tetracycline and cefazolin) [276].

## 6. Conclusions

*G. lucidum* bio-compounds are regarded as valuable in alternative cancer treatments based on non-natural products. The present work has compiled available data from various in vitro and in vivo studies on *G. lucidum* bio-compounds and their beneficial effects in anticancer treatment through their biological actions, such as anti-proliferative, antioxidant, immunomodulatory, anti-inflammatory, and anti-angiogenic effects. This paper presents information on the active bio-compounds in *G. lucidum* to obtain conclusive data and confirm their benefits regarding the mechanisms of anticancer action. Additionally, understanding the mechanisms of anticancer action, combined with other biological anticancer actions exerted by both the main bioactive compounds Gl-Ps and Gl-Ts and other compounds described in the paper (proteins, vitamins, metals, sterols, fatty acids, and nucleotide compounds), is necessary for targeted use in anticancer treatments. It is also crucial to comprehend that the biocomponents of *G. lucidum* are directly influenced by several factors, including the origin and culture medium, environmental conditions, temperature, humidity, and the quality of the environment from which the fungus originates. Therefore, further experimental, epidemiological, and clinical studies are needed to characterize the interactions of the administration of *G. lucidum* forms with different conventional anticancer drugs. More research is needed to combine *G. lucidum* bio-compound treatments with chemotherapy. Extensive pharmacological studies are also necessary to establish optimal dosages and assess the efficacy and safety of administration. Moreover, it is important to extend the research to identify metabolite subtypes that support the observed bioactivities, aiming to establish anticancer therapy procedures that promote general health and longevity.

## Figures and Tables

**Table 1:** Proximate composition of the fruiting body of *G. lucidum* from different country origins, reported as a percentage.

Mushroom Origin/ Constitutes	From Bangladesh	From Bangladesh	From Taiwan	From China	From Spain	From India	From Nigeria
Moisture %	12.19	47	-	-	-	7.5	2.78 ± 0.05
Ash %	3.93	6.3	1.8	1.21 ± 0.06	2.31 ± 0.12	18.7	8.42 ± 0.13
Water-soluble proteins %	28.6	19.50	7–8	7.47 ± 0.22	11.70 ± 0.35	23.6	16.79 ± 0.13
Total lipids %	2.4	3.00	3–5	-	-	5.8	1.52 ± 0.09
Fatty acid	-	-	-	1.44 ± 0.10	1.27 ± 0.09	-	1.22 ± 0.07
Total carbohydrates %	44.91	5.41	26–28	9.88 ± 1.04	11.02 ± 1.16	42.8	63.27 ± 0.20
Dietary fibers %	14.67	2.4	59	76.81 ± 3.46	69.35 ± 3.12	-	7.77 ± 0.34
References	[21]	[30,71]	[70]	[72]	[72]	[73]	[74]

**Table 2:** Structural characteristics, extraction, and fractionation techniques of polysaccharides from *G. lucidum*.

	Mushroom Part of *G. lucidum*	Monosaccharide Composition	Backbone	Extraction and Fractionation	Bioactivity	References
1	Fruiting body	Glucose, rhamnose glucose, galactose, rhamnose	β(1→3)-Glucan; -(1→3)-(1→4)-, (1→6)Heteroglycan α-(1→4), β-(1→6)-heteroglycan	Hot-water extraction; DEAE-cellulose and gel filtration; chromatography	Antioxidant activity	[94]
2	Fruiting body (cultivated)	Mannose, rhamnose, glucose, galactose	Heteroglucans (GLP, GLP1, GLP2, GLP3, GLP4) Main glycosidic bond	Ultrasonic extraction; Sevag method; ethanol precipitation; ultrafiltration membranes	Antioxidant activity in vitro by DPPH scavenging activity; reducing power; Fe^2+^ chelating activity; ORAC	[95]
3	Fruiting body (cultivated)	Glucose, galactose, mannose, arabinose	Heteropolysaccharide (GL-1; GL-V) (1→4)-galactan, Heteropolysaccharide	Soluble in water and in ethyl-acetate; Sevag method; dialysis	Bioactive compounds are an important source of anticancer agents	[96]
4	Extracellular	Galactose, mannose, glucose, arabinose, rhamnose	α-(1→4)-Galactose	DEAE-Sephcel and Sephadex G200.	Enhance T- and B-lymphocyte proliferation and antibody production	[97]
5	Fruiting body	Galactose, glucose, fucose	α-(1→6)-galactose α-(1→3)-Glucose	Hot-water extraction; DEAE-Sepharose Fast-Flow and Sephacryl S-300	Immunostimulatory activity of spleen lymphocyte proliferation	[98]
6	Fruiting body	Glucose, galactose, arhamnose	Heteroglycan α-(1→4), β-(1→6)	Hot-water extraction	Immunologically active; proliferation of B-lymphocytes with important immunologic activity	[99]
7	Fruiting body (cultivated)	Glucose, galactose, mannose, arabinose, xylose, fucose	Heteropolysaccharides glucans (1→3)-β-D-glucan with a few short (1→4)-linked glucosyl units	Extraction and separation of fractions with hot water, cold and hot 1 M NaOH	Antitumor activity against sarcoma solid tumor	[100]
8	Fruiting body	Galactose, glucose, fucose	α-(1→6)-, (1→2,6)-Galactose β-(1→3)-, (1→4,6)-Glucose	Hot-water extraction; DEAE-Sepharose Fast-Flow and Sepharose CL-6B	An immunostimulating potential	[101]
9	Fruiting body	Glucose, galactose, mannose	β-(1→3)(1→4)(1→6)-Glucan Heteropolysaccharides	Hot-water extraction; DEAE-cellulose-32 and Sephacryl S-200 h	Pronounced antioxidant activity in free radicals scavenging and Fe^2+^ chelating	[102]
10	Fruiting body (wild)	Galactose, rhamnose, and glucose in mole ratio of 1.00:1.15:3.22	Water-soluble polysaccharide α-(1→6)-, (1→2,6) Galactose β-(1→3)-, (1→4,6) Glucose	Hot water and ethanol precipitation; DEAE-Sepharose Fast Flow and Sephacryl S-300	Neutral heteropolysaccharide, which reported antihyperglycemia effects	[103]
11	Mycelium (cultivated)	Rhamnose, arabinose, mannose, glucose, galactose	Heteropolysaccharide α-D-Glc (1→6), α-D-Glc,α-D-Man (rhamnose and arabinose residues in the side chain)	Hot water; ethanol precipitation; Sevag method; dialysis	Antitumor activity against Human hepatocarcinoma cell line (HepG2) and tumor xenografts in ICR mice	[104]
12	Fruiting body	Glucose	Branched homo-glucan (GLP0; GLP1) (1→3)-β-D-glucan with (1→6)-β-D branches	Hot water followed by ethanol precipitation	Induced a cascade of immunomodulatory cytokines against sarcoma 180 solid tumor	[105]

**Table 3:** Minerals of *G. lucidum* fruiting body.

Elements	mg/100 g	mg/100 g	% or ppm	ppm
Potassium	432	3.590	1.11 ± 0.04 (%)	-
Phosphorus	225	4.150	30.17 ± 1.29 (ppm)	-
Sulfur	129	-	-	-
Magnesium	7.95	1.030	0.34 ± 0.01 (%)	50.76 ± 1.19
Sodium	2.82	375	229.88 ± 0.34 (ppm)	-
Calcium	1.88	832	1.99 ± 0.04%	-
Copper	27	-	7.43 ± 0.13 (ppm)	5.49 ± 0.35
Manganese	22	-	71.06 ± 1.56 (ppm)	20.19 ± 0.54
Iron	2.22	82.6	121.37 ± 1.82(ppm)	130.60 ± 1.63
Zinc	0.7	-	51.49 ± 2.16 (ppm)	8.45 ± 0.38
References	[30,71]	[153]	[74]	[156]

**Table 4:** Amino acids content in *Ganoderma lucidum* mushroom.

Amino Acid	Aspartic Acid	Threonine	Serine	Glutamic Acid	Proline	Glycine	Alanine	Valine
	Asp	Thr	Ser	Glu	Pro	Gly	Als	Val
mg AA/g protein	117	66	54	120	60	108	100	61
References	[27,184]	[27,184]	[27,184]	[27,184]	[27,184]	[27,184]	[27,184]	[27,184]
Amino acid	Methionine	Isoleucine	Leucine	Phenylalanine	Tyrosine	Histidine	Lysine	Arginine
	Met	Ile	Leu	Phe	Tyr	His	Lys	Arg
mg AA/g protein	6	36	55	28	16	12	21	22
References	[27,184]	[27,184]	[27,184]	[27,184]	[27,184]	[27,184]	[27,184]	[27,184]

**Table 5:** Compounds with antioxidant activity from *G. lucidum*.

Total Triterpenoids	Total Polysaccharides		Total Polyphenol Content (TPC)		Total Flavonoid Content (TFC)	Ascorbic Acid
/g d.w.	mg glucose equiv./g d.w.		mg/100 g d.w.		mg/100 g d.w.	mg/100 g d.w.
196.03–643.06	769.1	112.53	33.3–43.49	912.38	34.09–38.08	30.51–32.2
[188]	[192]	[188]	[21]	[192]	[21]	[21]

**Table 6:** The phenolic acids from *G. lucidum* [192].

Nr.	Phenolic Acids	Quantity (mg/100 g DW of Extract)
1	Tricaffeoyl-glucosyl-glucoside	13.54 ± 0.23
2	Tricaffeoyl-glucosyl	23.79 ± 0.24
3	Caffeoyltrihexoside	38.02 ± 0.30
4	Protocatechuic acid hexoside	19.09 ± 0.15
5	1-Caffeoylquinic acid	505.89 ± 3.21
6	trans-5-ꓑ-coumaroylquinic acid	0.46 ± 0.01
7	5-Caffeoylquinic acid	95.01 ± 0.92
8	Caffeoyl-2-hydroxyethane-1.1.2-tricarboxylic acid	213.89 ± 1.52
9	Yunnaneic acid F	1.29 ± 0.01
10	Salvianolic acid B	1.39 ± 0.01
	Sum	912.38 ± 20.14

Means ± SD (*p* ≤ 0.05; *n* = 3).

**Table 7:** Antioxidant capacity in the extract of G. lucidum.

DPPH		FRAP		ABTS
(%)	(µMol TE/g)	(μg/100 g)	(µMol TE/g)	(µMol TE/g)
24.04 ± 0.33	51.3 ±1.04	614.83 ± 0.05	49.87 ± 1.58	81.26 ± 1.10
[21]	[192]	[21]	[192]	[192]

Means ± SD (*p* ≤ 0.05; *n* = 3).

**Table 8:** Fatty acids content of two *Ganoderma* species [72].

Fatty Acids	*G. lingzhi* ± 15%	*G. lucidum* ± 15%
Total monounsaturated fatty acids	37.5	28.68
Total polyunsaturated fatty acids	43.84	49.93
Total saturated fatty acids	18.64	20.77
Total	99.98	99.38

**Table 9:** Anticancer activity of polysaccharides and triterpenes from *G. lucidum*.

	*G. lucidum* Compounds	Actions and Effects of Gl-Ps on Antitumor Activity	Mechanism of Action	References
1	Gl-Ps	Effects on dendritic cells	Gl-Ps acts on the maturation and function of cultured murine bone marrow-derived dendritic cells (DCs).	[211]
2	Gl-Ps	Effect on cytotoxicity	Gl-Ps acts with a specific T-lymphocyte cytotoxic (CTL) mechanism, which has been pulsed with the tumor antigen P815.	[212]
3	Gl-Ps	Evaluation of immunomodulatory effect on cytokines	Explain the mechanism of action on macrophages in which Gl-Ps (fractions) activate kinase to induce, in turn, activation of IL-1, IL-2, and TNF-α.	[213]
4	Gl-Ps	Effect of cytokine-induced killer cells (CIK)	Gl-Ps decreases the number of lymphokine-activated cytokines (LAK) and CIK-induced cytokine-killing cells.	[214]
5	Gl-Ps	Actions in immunopotentiation therapy against induced immunosuppression	Gl-Ps extract at low doses leads to increased immunological effector cell activity in immunosuppressed mice.	[215]
6	Gl-Ps	Effect on antioxidant enzyme activity	Gl-Ps from *G. lucidum* significantly reduced malondialdehyde (MDA) production and increased the activity of serum antioxidant enzymes in ovarian cancer therapy in rats.	[216]
7	Gl-Ps	Gl-Ps suppresses tumorigenesis, inhibits tumor growth	Gl-Ps affects immune cells, including B-lymphocytes, T-lymphocytes, dendritic cells, and natural killer cells. They are mediated by immunomodulatory, anti-angiogenic, and cytotoxic effects.	[217]
8	Gl-Ps	Antitumor effects by stimulating host immune function	Gl-Ps acts directly in activating lymphocytes that have been tested by incubating Gl-Ps with an antigen-deficient tumor cell line. Also, Gl-Ps acts on B16F10 melanoma cells.	[218]
9	Gl-Ps	Antitumor effects by stimulating host immune function	Gl-Ps can induce lymphocyte proliferation through action on B16F10 melanoma cells and IFN-γ production.	[219]
10	Gl-Ps	Antitumor activity manifested by a mixture of Gl-Ps and sulfates.	Gl-Ps sulfate showed remarkable inhibition of rat Heps proliferation.	[220]
11	Gl-Ps	Therapeutic potential in inflammatory breast cancer (IBC).	Study results provide evidence that Gl-Ps treatment suppresses protein synthesis and tumor growth by affecting survival signaling pathways in mice injected with IBC cells, suggesting a natural therapeutic potential for breast cancer.	[221]
12	Gl-Ps	The ability of isolated Gl-Ps fractions (F3) to induce innate inflammatory cytokines	Enhanced Th1 response with high levels of IFN-γ and IL-2. Cell wall Gl-Ps were inducers of innate inflammatory cytokines, and extracellular Gl-Ps demonstrated a high capacity to modulate cytokine responses to IL-17 production.	[222]
13	Gl-Ps	Potential anticancer activity	They discussed the mechanisms of anticancer activity attributed to Gl-Ps by highlighting immunomodulatory, anti-proliferative, pro-apoptotic, antimetastatic and anti-angiogenic effects.	[223]
14	Gl-Ps	Antitumor action and immunomodulatory effects of Gl-Ps in rats	Gl-Ps increased the serum concentration of Il-2, INF-γ and tumor necrosis factor-α. It increased the cytotoxic activity of natural killer cells and T cells and led to prolonged lifespan of brain glioma-bearing rats.	[224]
15	Gl-Ps, spores	Antitumor action of a novel polysaccharide with an estimated average molecular weight of 1.5 × 104 Da	In vivo antitumor activity tests showed that Gl-Ps could significantly inhibit S180 tumor growth in mice. No drug-related toxic reactions were observed.	[225]
16	Gl-Ts	Ganoderic acids from spores and their cytotoxicity	The cytotoxicity of the compounds isolated from the *Ganoderma* spores was carried out in vitro against Meth-A and LLC tumor cell lines.	[227]
17	Gl-Ts	Anticancer study of lucialdehydes B, C (2,3), ganodermanonol, and ganodermanondiol	Cytotoxic mechanism. Lucialdehyde C exhibited the most potent cytotoxicity against CLL, T-47D, sarcoma 180, and Meth-A tumor cells.	[227]
18	Gl-Ps Gl-Ts	Antitumor effect of aqueous extract; cytotoxic activity of alcoholic extract	Manifestation of a significant antitumor effect in several tumor-bearing animals; manifestation of an anti-angiogenic effect that may be involved in the antitumor activity.	[228]
19	Gl-Ts	Ability of ganoderic acid X (GAX) to inhibit topoisomerases and interfere with apoptosis	Mechanisms of chromosomal DNA degradation, cancer cell apoptosis, mitochondrial membrane disruption, and caspase-3 activation have been elucidated upon GAX treatment of HuH-7 human hepatoma cells.	[229]
20	Gl-Ts	Cytotoxicity of GA-T on different human carcinoma	It was shown in vivo to significantly inhibit proliferation of lung cancer cells by inducing apoptosis by GA-T	[230]
21	Gl-Ts	The effect of ganoderic acids A, F, and H on breast cancer cells was evaluated	GA-A, GA-F, and GA-H suppressed cell proliferation, colony formation, and invasive behavior of MDA-MB-231 cells. They have biological effects by inhibiting transcription factors AP-1 and NF-κB.	[231]
22	GA-T	Studies of anti-invasive and antimetastatic mechanisms of GA-T in vitro in lung cancer	GA-T dose-dependently inhibited 95-D cell migration by wound healing assay, promoting cell aggregation and inhibiting cell adhesion to the extracellular matrix (ECM). GA-T prevents tumor metastasis in highly metastatic lung carcinoma.	[232]
23	Gl-Ts.	Anticancer, anti-inflammatory, and antimetastatic activities of *G. lucidum* extracts	Gl-Ts from *G. lucidum* reduces the production of IL-8, IL-6, MMP-2, and MMP-9 in breast cancer and melanoma cells. They decrease cancer cell viability in a time and dose-dependent manner.	[233]
24	GL-Ts	Investigation of Gl-Ts with activity in inhibiting growth of pulmonary carcinoma metastates and suppressing colonic inflammation	The triterpene extracts exhibit inhibitory activity against foodborne carcinogen-induced mouse colon carcinogenesis. All suppressive functions were enhanced by high doses of triterpene extract.	[234]
25	Gl-Ts NTF, ATF	Evaluation of anticancer effects of NTF (neutral triterpene fraction) and ATF (acidic triterpene fraction) on human colorectal cancer	The cytotoxic effects of Gl-Ts on human colon cancer cells SW480, SW620, SW116, and mouse embryonic fibroblast cells NIH3T3 were studied. Compounds isolated from NTF acted as antitumorals by inducing apoptosis.	[235]
27	GlSO	Mechanistic investigation of the anticancer-gene effect of GlSO (*G. lucidum* spore oil) on mammary cancer cells	Growth of MDA—MB-231 cells, in vitro, were inhibited by treatment with GlSO (0.2, 0.4, and 0.6 µL/mL). In vitro, GlSO increased Bax and caspase-3 expression but did not affect caspase-8 expression.	[236]
27	Gl-Ts	Anticancer potential of *G. lucidum* against prostate cancer (PC-3)	*G. lucidum* has been shown to prevent prostate cancer cell growth and stimulate apoptosis in PC-3 cells by preventing STAT-3 translocation (signal transduction and activation of transcription).	[237]
28	Gl-Ts	Effects on colorectal cancer. Involves suppression of NF-κB-regulated inflammation and carcinogenesis	In vitro administration of GLSF extract at non-toxic concentrations to mice inoculated with CT27 tumor cells significantly potentiated paclitaxel-induced growth inhibition and apoptosis in CT27 and HCT-15 cells.	[238]
29	Gl-Ps, Gl-Ts	Evaluation of the effects on skin carcinogenesis analyzed on JB6 cells in SKH-1 mice	Reduced incidence and multiplicity of skin tumors. In tumor-free skin tissue of mice, Gl-Ps and Gl-Ts attenuated UV-induced epidermal thickening. Gl-SF increased CD8 and Granzyme B expression.	[239]

## Data Availability

Data is contained within the article.

## Abbreviations

Gl-Ps	*G. lucidum* polysaccharides	ROS	Reactive Oxygen Species
Gl-Ts	*G. lucidum* triterpenoids	GA-A	Ganoderic acids A
TPC	Total polyphenol content	GA-F	Ganoderic acids F
TFC	Total flavonoid content	GA-H	Ganoderic acids H
IL	Interleukin	GA-T	ganoderic acid T
TNF-α	Tumor Necrosis Factor Alpha	GA-Me	Ganoderic acid Me
INF-γ	Interferon Gamma	MMP	Matrix metalloproteinase;
TGF-α	Transforming Growth Factor-Alfa	TCL	T-lymphocyte cytotoxic
TGF-β	Transforming Growth Factor-Beta	NK	Natural killer cells;
VEGF	Vascular Endothelial Growth Factor	PKC	Protein kinase C
NO	Nitrogen species	ECM	Extracellular matrix;
MDA	Malondialdehyde	DCs	Dendritic cells
HPLC-ESI-MS	Liquid chromatography coupled with electrospray ionization mass spectrometry	TEAC	Trolox equivalent antioxidant capacity
